# Effects of the coupling of dielectric spherical particles on signatures in infrared microspectroscopy

**DOI:** 10.1038/s41598-022-16857-1

**Published:** 2022-08-03

**Authors:** Beibei Kong, Maren Anna Brandsrud, Johanne Heitmann Solheim, Ingrid Nedrebø, Reinhold Blümel, Achim Kohler

**Affiliations:** 1 Faculty of Science and Technology, Norwegian University of Life Sciences, Ås, Norway; 2 Department of Physics, Wesleyan University, Middletown, Connecticut USA

**Keywords:** Infrared spectroscopy, Optical spectroscopy, Biotechnology, Biological physics

## Abstract

Infrared microspectroscopy is a powerful tool in the analysis of biological samples. However, strong electromagnetic scattering may occur since the wavelength of the incident radiation and the samples may be of comparable size. Based on the Mie theory of single spheres, correction algorithms have been developed to retrieve pure absorbance spectra. Studies of the scattering characteristics of samples of different types, obtained by microspectroscopy, have been performed. However, the detailed, microscopic effects of the coupling of the samples on signatures in spectra, obtained by infrared microspectroscopy, are still not clear. The aim of this paper is to investigate how the coupling of spherical samples influences the spectra. Applying the surface integral equation (SIE) method, we simulate small dielectric spheres, arranged as double-spheres or small arrays of spheres. We find that the coupling of the spheres hardly influences the broad oscillations observed in infrared spectra (the Mie wiggles) unless the radii of the spheres are different or the angle between the direction of the incident radiation and the normal of the plane where the spheres are located is large. Sharp resonance features in the spectra (the Mie ripples) are affected by the coupling of the spheres and this effect depends on the polarization of the incident wave. Experiments are performed to verify our conclusions.

## Introduction

Infrared microspectroscopy imaging techniques are useful tools for the analysis of biological samples^1–3^. According to the absorbance spectra obtained by infrared microscopy, the chemical composition of the sample can be evaluated since different molecular bonds absorb light with a specific wavelength^4^. Thus, via this technique, the analysis of the biochemical changes inside cells and tissues can be performed without perturbing the sample, which can help improve the sensitivity and specificity of the recognition of samples^1^.

Strong molecular absorption bands occur in the mid-infrared spectral region with wavelengths ranging from 2.5 to $12.5\, \mathrm {\upmu }$m. Coincidentally, this wavelength range is (within a small factor) also the typical size range of biological materials, typically investigated via mid-infrared spectroscopy^1^. For example, human cells typically range between 10 and $30\, \mathrm {\upmu }$m^4,5^ and pollen grains range in size between 10 and $40\, \mathrm {\upmu }$m^6^. This is also the size range of polymethyl-methacrylate (PMMA) micro-spheres that have recently been investigated as model systems for biological cells^6,7^. In these cases, where the wavelength of the incident electromagnetic radiation is comparable to the size of the samples, strong electromagnetic scattering occurs. For spherically- or spheroically-shaped samples, such as pollen samples^6^ or PMMA spheres^6,7^, this scattering can be interpreted as Mie-type scattering^5^. The scattering signature is entangled with absorption signatures in the absorbance spectra, increasing the difficulty of the quantification of the pure chemical absorption properties of the material. Although many correction algorithms of the infrared spectra have been developed and proved to have good performance^8,9^, these algorithms are all based on van de Hulst’s Mie theory of single spherical samples^10^. Moreover, the scattered field is expected to provide physical and chemical information of the sample. Thus, the study of the scattering characteristics of different types of samples is very important.

Some studies have been performed on the interpretation of scattering characteristics of infrared microscopy spectra of biological samples^11,12^. Since biological cells or tissues are not always spherical samples, the effect of the deformation of biological samples on the Mie-type signatures and the chaotic scattering caused by sample deformation has been studied in^11^. It is also very common that the sample is not a single particle. The effects of the coupling of the samples on the spectra are therefore important to understand. As a first stab into this direction, the authors of^12^ investigated absorption, scattering, and extinction spectra of random clusters of mono- and poly-dispersed PMMA spheres with radii in the range from 2.5 to $10\, \mathrm {\upmu }$m. The following important results were obtained. (1) Electromagnetic coupling between different spheres in random clusters is important. (2) The coupling effects depend on the packing fraction $\phi =V_{sp}/V_{box}$ in the cluster, where $V_{sp}$ is the total volume occupied by the spheres in a box of volume $V_{box}$. (3) There is a marked transition in the qualitative appearance of IR spectra at the critical packing fraction of $\phi \approx 0.18$, which, importantly, coincides with the percolation threshold for a hard-spheres gas. (4) As $\phi$ increases, absorption spectra approach the spectra of bulk material. (5) Delicate features of the spectra of single spheres, such as resonances, Mie wiggles and Mie ripples^10^ tend to get averaged away in poly-dispersed clusters. The authors of^12^ predicted these results on the basis of super-computer *T*-matrix^13^ simulations and confirmed these results experimentally. While these results are important for analyzing spectra of randomly distributed clusters in biological systems and colloidal suspensions, we are interested here in the regime between the extremes of single spheres and the statistical limit of many randomly distributed scatterers. This few-particle regime is the most difficult regime to investigate since the number of particles is at the limit of individual-particle methods but the number of particles is not yet large enough to employ statistical methods, e.g., averaging of spectra, as done in^12^. In particular, we are interested in the details of how two spheres, or small clusters of spheres, exactly interact with each other, how they are electromagnetically coupled, and how this coupling diminishes with the distance between scatterers. Consequently, in the following, we investigate to what extent the scattering at one cell (modeled as a sphere) is affected by the presence of neighboring cells (also modeled as spheres). In contrast to the many-body context^12^, and taking into account the typical sizes and shapes of cells and the optical apertures employed in biological and medical IR applications, in our few-body context, and for scatterers arranged in a plane orthogonal to the direction of the incident beam, a strong coupling effect might not be expected. This is so, since a ray that hits a neighboring cell is necessarily a scattered ray that has already been accounted for in the scattering off a single cell. It may only be “cancelled” as a scattered ray, and therefore modify the single-cell scattering signature, if a neighboring cell redirects this scattered ray into the forward direction with zero phase shift. This process is very unlikely, pointing to a small coupling effect. In contrast, as we show below, for scatterers (cells) ovelapping in beam direction, significant changes in scattering occur compared to the case of a single scatterer. However, in general, and without numerical support, it is not clear in what way and by how much the coupling of samples affects the spectral features in infrared microspectroscopy. Since cells and other scatterers in close proximity is a common situation we meet in infrared transmission measurements, it is important to address these questions both quantitatively and qualitatively.

In this paper, we investigate the scattering of double-spheres and sphere arrays for perpendicular and oblique incidence with respect to the plane in which the spheres are located. Oblique incidence is interesting to consider as infrared microscopes have high numerical apertures. The surface integral equation (SIE) method^14^, which is a full-wave numerical method for solving Maxwell’s equations, is used to provide accurate scattered signals of the targets. The effects of the coupling of dielectric spherical particles on both the wiggles and the ripples of signatures in infrared microspectroscopy are investigated. Our results show that, as long as all scatterers are arranged in a plane orthogonal to the direction of the incident beam, the wiggles do not experience a significant change whether the sample contains one or several spheres. This is good news for the correction algorithms of infrared spectra^8,9^. For the correction algorithms, the wiggle structure is very important. And in many biological samples, ripples do not appear and they are therefore not important for the correction. The current correction algorithms will be valid no matter if we have one or several spheres. Experimental results are provided to validate our conclusions.

## Definitions and methods

### The extinction efficiency

We consider electromagnetic scattering by an arbitrary-shaped object with surface *S*. The object is illuminated by an incident wave $(\mathbf {E}^\mathrm{i}, \mathbf {H}^\mathrm{i})$. Let $(\mathbf {E}^\mathrm{s}, \mathbf {H}^\mathrm{s})$ denote the field scattered by the object. The total electric and magnetic fields can be expressed as ${\mathbf {E}}={\mathbf {E}^\mathrm{i}}+{\mathbf {E}^\mathrm{s}}, \mathbf {H}={\mathbf {H}^\mathrm{i}}+{\mathbf {H}^\mathrm{s}}$, respectively. The total cycle-averaged power flowing into the object and the power scattered from the object can be expressed as

1 $$\begin{aligned}{}&{P_\mathrm{abs}} =-\int _{s}{\frac{1}{2} Re\left\{ \hat {\mathbf{n}} \left( \mathbf {r}\right) \cdot \left[ \mathbf {E}^{*} \left( \mathbf {r}\right) \times \mathbf {H} \left( \mathbf {r}\right) \right] \right\} }dS , \end{aligned}$$

2 $$\begin{aligned}{}&{P_\mathrm{sca}} =\int _{s}{\frac{1}{2} Re\left\{ \hat {\mathbf{n}} \left( \mathbf {r}\right) \cdot \left[ \mathbf {E}^{s*}\left( \mathbf {r}\right) \times \mathbf {H}^{s}\left( \mathbf {r}\right) \right] \right\} }dS , \end{aligned}$$

where $\hat {\mathbf{n}} \left( \mathbf {r}\right)$ is the outward-pointing unit normal vector at a point $\mathbf {r}$ on *S*. Let ${I_\mathrm{inc}}$ be the incident flux of radiative energy. Then, the absorption and scattering cross sections can be expressed as $\sigma _\mathrm{abs,\mathrm sca}=P_\mathrm{abs,\mathrm sca}/{I_\mathrm{inc}}$. The total cross section, which is commonly called the extinction cross section, is the sum of the absorption and scattering cross sections, i.e., $\sigma _\mathrm{ext}=\sigma _\mathrm{abs}+\sigma _\mathrm{sca}$. The extinction efficiency $Q_\mathrm{ext}$ can be obtained by dividing the extinction cross section by the geometrical cross section *g* of the scatterer, i.e., $Q_\mathrm{ext}= \sigma _\mathrm{ext}/g$.

For spherical scatterers of radius *R*, the extinction efficiency can be analytically computed according to the Lorentz–Mie theory, as

3 $$\begin{aligned} Q_\mathrm{ext} =\frac{2}{x^2}\sum _{n=1}^{\infty }\left( 2n+1\right) Re\left\{ a_n+b_n\right\} , \end{aligned}$$

where $x=2\pi R/\lambda$ is the size parameter of the sphere, $\lambda$ is the free-space wavelength, and $a_n$ and $b_n$ are the electric and magnetic scattering coefficients^10^, respectively.

### Infrared absorbance signature in microspectroscopy

In our infrared transmission measurements, the sample is illuminated by an infrared beam of intensity $I_0$. After being scattered and absorbed by the sample, the infrared beam hits the detector of geometrical cross section *G* with intensity *I*. We assume that *G* is much larger than *g*. The apparent absorbance, defined as

4 $$\begin{aligned} A=-\log _{10}\left( \frac{I}{I_0}\right) , \end{aligned}$$

is measured.

According to the conservation of energy, we have

5 $$\begin{aligned} I_0 G=I_0 \sigma _\mathrm{ext}+IG=I_0 gQ_\mathrm{ext}+IG . \end{aligned}$$

The apparent absorbance can then be expressed in terms of the extinction efficiency, as

6 $$\begin{aligned} A=-\log _{10}\left( 1-\frac{g}{G} Q_\mathrm{ext}\right) . \end{aligned}$$

Thus, the measured quantity *A*, obtained in infrared microspectroscopy, is related to the extinction efficiency. In our numerical simulations, we calculate the extinction efficiency of the scatterers to investigate the effect of the coupling of the dielectric spheres on the signature of infrared microspectroscopy^7^.

### Surface integral equation method

The extinction efficiency of the dielectric spheres is computed with the SIE method^14^. As one of the most powerful numerical methods in computational electromagnetics, the SIE method is widely used to provide accurate solutions for many electromagnetic problems. Instead of solving the original Maxwell equations in the entire 3D space, the SIE method solves the equivalent currents on the surfaces and the interfaces of the targets according to the surface equivalence principle. Compared with volume-based methods such as the finite element method (FEM)^13^ and the finite-difference time-domain (FDTD) method^13^, whose number of unknowns increases with the third power of the dimension of the target, the SIE method has significantly fewer unknowns, which increase with the second power of the dimensions of targets. The SIE method is thus particularly suitable for the simulation of homogeneous dielectric targets considered in this paper. The extinction efficiency can be obtained directly from the excitation vector of the SIE matrix equation and the associated solution vector, as explained in^15,16^.

For sphere arrays we considered, the size of the targets can reach up to $16.5\, \mathrm {\upmu }$m. At the short-wavelength end of our simulations (at about $1.7\, \mathrm {\upmu }$m), a target size of $16.5\, \mathrm {\upmu }$m is quite large and poses a challenge for our numerical simulations. Thus, in order to reduce the simulation time and memory requirement, the Multilevel Fast Multipole Algorithm (MLFMA)^17^ is used to accelerate the computation of the matrix-vector multiplications. To further increase the efficiency of the numerical method, the near-field matrix of MLFMA is used as a preconditioner to speed up the iterative convergence^18^.

### Infrared spectral acquisition

Spheres made of Poly(methyl methacrylate) (PMMA), with a diameter of $5.5\, \mathrm {\upmu }$m, were measured using a Vertex 70 FTIR spectrometer coupled with a Hyperion 3000 microscope (both Bruker Optik, Ettlingen, Germany). The measurements were recorded at 15$\times$ magnification with a numerical aperture of 0.4, using a fully open aperture and a mercury cadmium telluride focal plane array (MCT-FPA) detector. The spheres were placed on 1 mm thick CaF_2_ microscope slides, using perfluoronoane as a dispersing agent. Spectra were recorded in the range 899–3844 cm$^{-1}$, with a spectral resolution of 8 cm$^{-1}$ and digital spacing of 3.86 cm$^{-1}$. 132 scans were averaged for each spectrum. Two images were collected from each slide, from in total 3 slides. Then spectra from single spheres and double-spheres were selected manually. These spectra were first preprocessed using multiplicative signal correction (MSC) to standardize the spectra with respect to scaling and constant offset, and then averaged.

## Results

In this section, we investigate the effect of the coupling of spherical scatterers on the apparent absorbance. The extinction efficiency of dielectric spheres is calculated using the SIE method. We first calculate the extinction efficiency of double-spheres and thereafter analyze the effect of the distance of the spheres and that of the polarization and incident angle of the incident wave. Then we calculate and analyze the extinction efficiency of sphere arrays. Finally, we compare the simulation results of PMMA spheres with the measured results.

### Qext of two touching spheres

**Figure 1:**
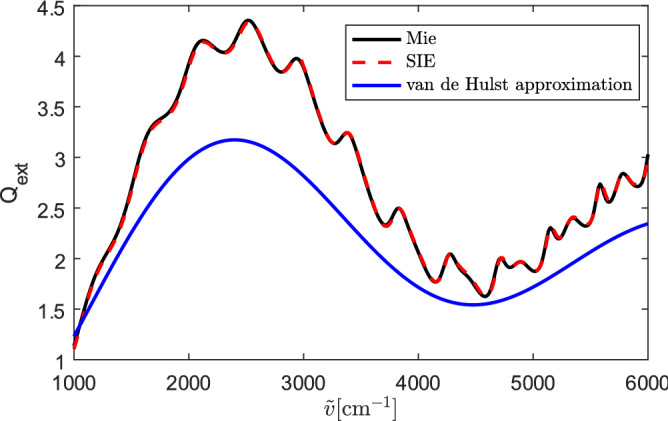
Extinction efficiency of a single sphere with a diameter of $5.5\, \mathrm {\upmu }$m and a refractive index of 1.49, obtained by exact Mie theory (black line), compared with our numerical SIE method (red dashed line). Blue line: analytical van de Hulst result (7).

First, we calculate the scattering by a single PMMA sphere with an approximate constant refractive index. The diameter of the sphere is $5.5 \, \mathrm {\upmu }$m. The extinction efficiency of the sphere is calculated by the SIE method. The wavenumber ($\tilde{v}=1/\lambda$) ranges from $\tilde{v}=1000$ cm$^{-1}$ to $\tilde{v}=6000$ cm$^{-1}$. In this transition region, we use $n=1.49$ as the approximate real part of the refractive index of the PMMA sphere. The imaginary part of the index of refraction is set to zero. The numerical results together with the analytical Mie solution are shown in Fig. 1. The results obtained by the SIE method agree well with the exact Mie solution. Also shown in Fig. 1 is $Q_\mathrm{ext}$ according to van de Hulst’s analytical theory of forward scattering^10^, which considers neither diffraction nor refraction and takes only straight-through rays into account. Defining $\rho =2\pi R\tilde{\nu }(n-1)$, van de Hulst’s analytical expression for $Q_\mathrm{ext}$ of a sphere is^10^

7 $$\begin{aligned} Q_\mathrm{ext} = 2 - \frac{4}{\rho } \sin (\rho ) + \frac{4}{\rho ^2} [1-\cos (\rho )]. \end{aligned}$$

This result is shown as the blue line in Fig. 1. While, compared with the exact result, the amplitude of (7) is a bit off (not surprising in the absence of including refraction and diffraction effects), the frequency of (7) is very well reproduced. This is due to the fact that the wiggles, described by (7), are due to interference of the incident radiation with the scattered radiation in *forward* direction, which is clearly independent of diffraction and refraction effects, which both result in radiation scattered out of the forward direction.

**Figure 2:**
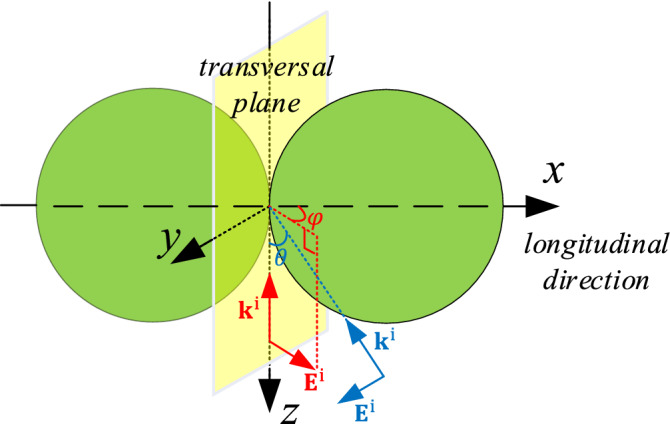
Geometry of the scattering problem of the double-spheres. $\mathbf{k}^i$ denotes the incident direction. $\mathbf {E}^\mathrm{i}$ is the incident electric field.

**Figure 3:**
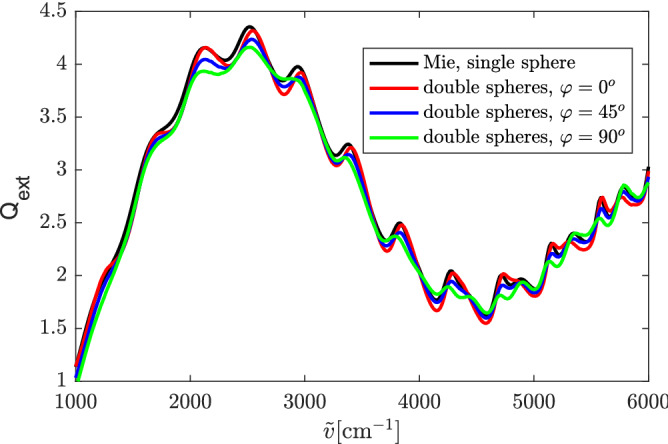
Extinction efficiency of the double-sphere for various polarization angles $\varphi$, obtained by our SIE method, compared with the Mie solution of the single sphere. The spheres have a diameter of $5.5\, \mathrm {\upmu }$m and a refractive index of 1.49.

Next, we calculate the extinction efficiency of two touching spheres, as shown in Fig. 2. Both spheres have a diameter of $5.5\, \mathrm {\upmu }$m and a refractive index of 1.49. The double-sphere is illuminated by a plane wave propagating along the negative *z* direction. The angle between the polarization direction of the incident electric field (see the red arrow in Fig. 2) and the *x* axis is $\varphi$. The extinction efficiencies of the double-sphere for $\varphi =0^\circ$, $45^\circ$, and $90^\circ$ are compared with the analytical Mie solution of the single sphere, as shown in Fig. 3. It can be observed that $Q_\mathrm{{ext}}$ of the double-sphere has the same wiggle structure (the long-range oscillations) as $Q_\mathrm{{ext}}$ of the single sphere (see Fig. 1). The coupling of the two touching spheres only affects the amplitude of the ripples (the sharp peaks) of $Q_\mathrm{{ext}}$ but hardly affects the locations of the ripples.

**Figure 4:**
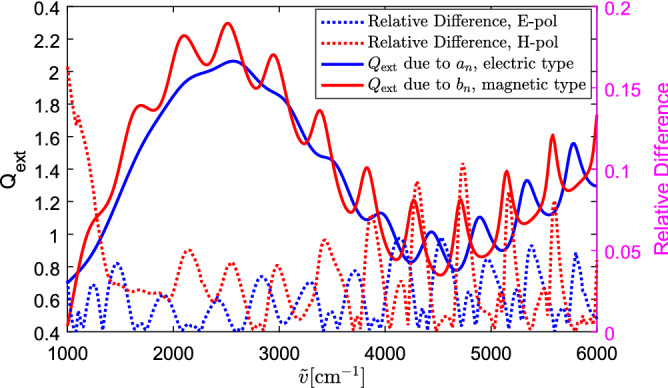
Contributions of $a_n$ and $b_n$ to $Q_\mathrm{ext}$ of the exact Mie solution and the relative difference between $Q_\mathrm{ext}$ of the double-sphere and the single sphere in Fig. 3.

In order to quantitatively investigate the effect of the polarization of the incident wave, we calculate the relative difference between the extinction efficiency of the double-sphere and the single sphere, defined as

8 $$\begin{aligned} \delta =\sqrt{\frac{\sum _{i=1}^{K}\left| Q_\mathrm{ext, d}\left( i\right) -Q_\mathrm{ext, s}\left( i\right) \right| ^2}{\sum _{i=1}^{K}\left| Q_\mathrm{ext, s}\left( i\right) \right| ^2}} , \end{aligned}$$

where $Q_\mathrm{ext, d}$ denotes the extinction efficiency of the double-sphere calculated by the SIE method, $Q_\mathrm{ext, s}$ the extinction efficiency of the single sphere obtained by the SIE method, and *K* the number of wavenumbers. The relative difference $\delta$ of $Q_\mathrm{ext, s}$ and $Q_\mathrm{ext, d}$ under the three polarization angles $\varphi =0^\circ$, $45^\circ$, and $90^\circ$ are $\delta =0.021,0.022,0.037$, respectively. The difference between the extinction efficiency of the single sphere and the double-sphere is larger when the electric field is along the transversal plane than when the electric field is along the longitudinal direction. Since the extinction efficiency of a sphere can be calculated by adding the contributions of the electric ($a_n$) and magnetic ($b_n$) scattering coefficients^10^, we plot the contributions of $a_n$ and $b_n$ to $Q_\mathrm{ext}$ separately^7^ (see Fig. 4). In Fig. 4, we can clearly see that the magnetic-type resonances, i.e., the ripples that are due to the maxima of the $b_n$, are stronger than the electric-type resonances which arise from $a_n$ (see also^7^). Plotting the relative difference between the extinction efficiency of the double-sphere and the single sphere at each point of wavenumbers (Relative Difference$(\tilde{v})=\left| \frac{Q_\mathrm{ext, d}\left( \tilde{v}\right) -Q_\mathrm{ext, s}\left( \tilde{v}\right) }{Q_\mathrm{ext, s}\left( \tilde{v}\right) }\right|$) in Fig. 4, we observe that when the incident electric field is along the longitudinal direction (we call it E-polarization), the difference of $Q_\mathrm{ext}$ between the double-sphere and the single sphere is much greater near the electric-type resonances, and vice versa.

**Figure 5:**
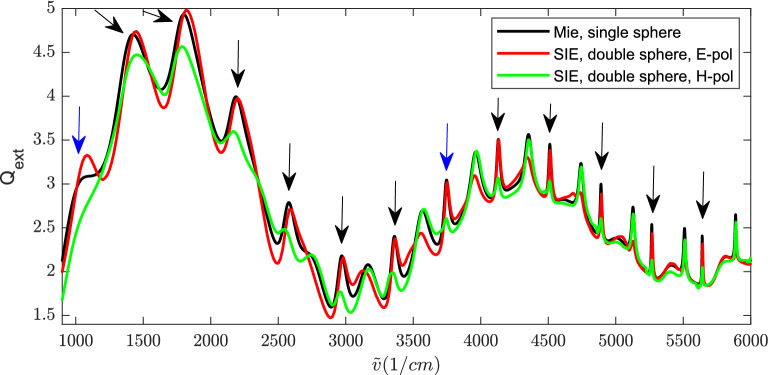
Extinction efficiency of the double-sphere, obtained by our numerical SIE method, compared with the exact Mie solution of the single sphere. The spheres have a diameter of $5.5\, \mathrm {\upmu m}$ and a refractive index of $\sqrt{3}\approx 1.7321$. In this figure, the ripples of the single sphere that are marked by arrows are magnetic-type resonances, and the other ripples are electric-type ones.

To further investigate the effect of the coupling of two touching spheres on the ripples, we calculate the scattering of spheres for a larger refractive index *n*. This has advantages, since a larger refractive index results in stronger Mie resonances. For the following simulations, we chose spheres with a relative dielectric constant $\epsilon =3$, which results in a refractive index of $n=\sqrt{\epsilon }\approx 1.7321$. For this case, we calculated the extinction efficiency of two touching spheres for different polarizations and compared with the Mie result for a single sphere. The diameter of each sphere is $5.5\, \mathrm {\upmu m}$. Again, we observe that $Q_\mathrm{{ext}}$ of the double-sphere has almost the same wiggle structure as a single sphere. The coupling effect of the two spheres is significant for the electric-type resonances when the incident field is E-polarized. For the H-polarized incident wave (the incident magnetic field is along the longitudinal direction), the coupling of the two spheres mainly affects the amplitudes of the magnetic-type ripples (see Fig. 5).

**Figure 6:**
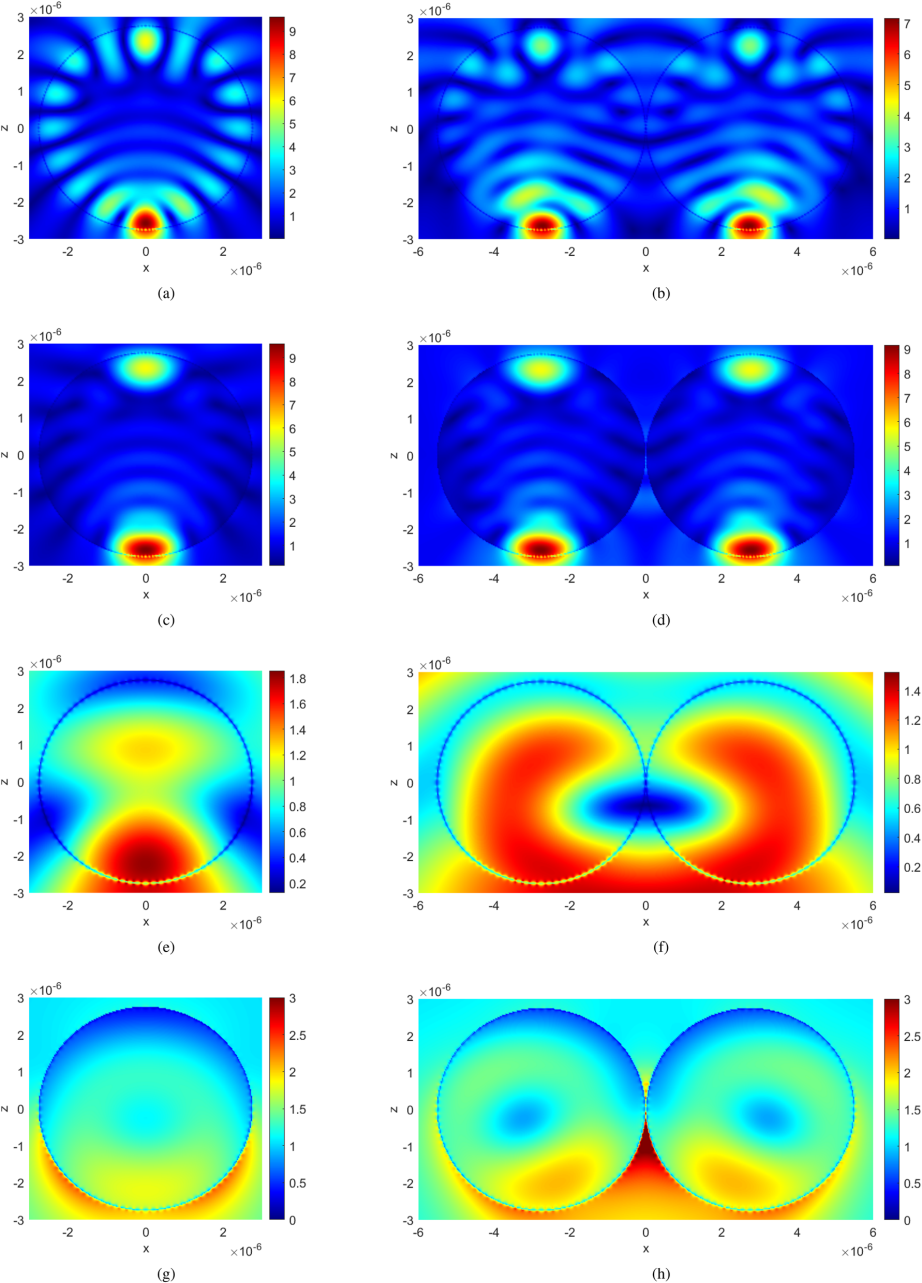
The absolute value of the electric field at two different wavenumbers ($\tilde{v}=3747\, \mathrm {cm}^{-1}$ and $\tilde{v}=1051\, \mathrm {cm}^{-1}$) comparing the fields of a single sphere with the case a touching double-sphere for *x*- and *y*-polarization. (**a**) Single sphere at $\tilde{v}=3747 \,\mathrm {cm}^{-1}$ (*y*-polarized). (**b**) Double-sphere at $\tilde{v}=3747 \,\mathrm {cm}^{-1}$ (*y*-polarized). (**c**) Single sphere at $\tilde{v}=3747 \,\mathrm {cm}^{-1}$ (*x*-polarized). (**d**) Double-sphere at $\tilde{v}=3747 \,\mathrm {cm}^{-1}$ (*x*-polarized). (**e**) Single sphere at $\tilde{v}=1051 \,\mathrm {cm}^{-1}$ (*y*-polarized). (**f**) Double-sphere at $\tilde{v}=1051 \,\mathrm {cm}^{-1}$ (*y*-polarized). (**g**) Single sphere at $\tilde{v}=1051 \,\mathrm {cm}^{-1}$ (*x*-polarized). (**h**) Double-sphere at $\tilde{v}=1051\, \mathrm {cm}^{-1}$ (*x*-polarized).

The electric near-fields of the single sphere and those of the double-sphere for the wave propagating in the $-z$ direction, corresponding to a sharp resonance (ripple) at $\tilde{v}=3747$ cm$^{-1}$ (magnetic-type resonance, marked by the blue arrow in Fig. 5), are shown in Fig. 6(a)–(d), where the absolute value of the electric field, $\left| \mathbf {E}\right| =\sqrt{\mathbf {E}\cdot \mathbf {E}^{*}}$, is shown in the *x*-*z* plane. The incident electric field is *y*-polarized for the results in Fig. 6(a)-(b), and it is *x*-polarized for the results in Fig. 6(c)-(d). Figure 6(a) shows the standing wave inside the surface of the sphere corresponding to the ripple at $\tilde{v}=3747$ cm$^{-1}$ , guided along the intersection of the sphere’s surface and the *x*-*z* plane. This is a typical whispering-gallery mode structure^7^. In Fig. 6(b), the whispering-gallery mode structure in both spheres is significantly affected since the neighbor sphere touches the surface where the standing wave is guided. When the incident electric field is *x*-polarized, the standing wave appears along the *y*-*z* plane that is parallel with the incident magnetic field, as shown in Fig. 6(c). For the double-spheres arranged along the *x* axis, as shown in Fig. 6(d), the standing waves in each sphere are hardly affected by the adjacent sphere. Comparing Fig. 6(c) and (d), we see that the distribution of the electric field of the double-sphere is very similar to that of the single sphere.

We also show the results at a much lower wavenumber. In Fig. 6(e)-(f) and (g)-(h), we plot the electric fields of the single and double spheres at $\tilde{v}=1051$ cm$^{-1}$ (see the blue arrow in Fig. 5) for the incident plane wave propagating along the negative z direction with *y*- and *x*-polarized electric field, respectively. Since the resonance at $\tilde{v}=1051$ cm$^{-1}$ is of magnetic-type, we can again observe from Fig. 6(e)-(f) that the coupling of the double spheres significantly affects the distribution of the electric field when the incident magnetic field is along the longitudinal direction (*x*-axis). Comparing the results in Fig. 6(g)-(h) with the results in Fig. 6(c)-(d), we also observe that the coupling between the spheres is stronger at larger wavelengths. The spheres are more decoupled at shorter wavelengths.

### Qext of two spheres with different distance

**Figure 7:**
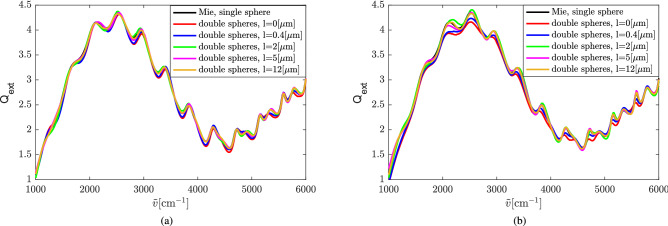
Extinction efficiency of the double-sphere with different *l* under (**a**) E-polarization and (**b**) H-polarization.

Next, we increase the distance between the two spheres, as sketched in Fig. 2, from $0\, \mathrm {\upmu }$m (when the spheres are touching) to $12\, \mathrm {\upmu }$m. This distance is denoted by *l*. Both spheres have a diameter of $5.5\, \mathrm {\upmu }$m and a refractive index of 1.49. The extinction efficiencies of the double-sphere with different *l* under E- and H-polarization are shown in Fig. 7(a) and (b), respectively. The change of the distance between the two spheres does not seriously affect $Q_\mathrm{{ext}}$.

**Figure 8:**
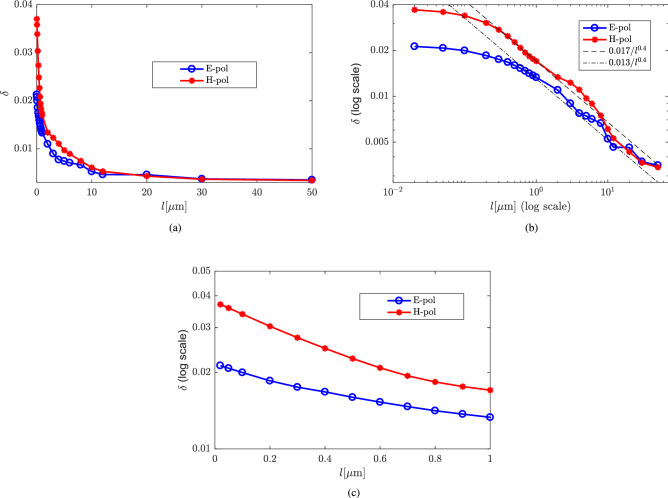
Relative difference $\delta$ defined in (Eq. 8) between the extinction efficiency of the double-sphere, obtained by our SIE method, and the single sphere, obtained via the exact Mie solution, as a function of the distance *l* between the two spheres. The spheres have a diameter of $5.5\, \mathrm {\upmu }$m and a refractive index of 1.49. (**a**) The numerical data for $\delta$ on a linear scale. (**b**) The data of (**a**) but on a log-log scale. This view of the data brings out the asymptotic power-law decay of $\delta$ at large distances *l* of the two spheres. It also shows that there is a cross-over of the decay from exponential at small distances (see (**c**)) to power-law at large distances of the two spheres. The two power-law dashed lines are drawn to guide the eye. (**c**) The data of (**a**) but on a semi-log scale. This view of the data brings out the exponential decay of $\delta$ in the evanescent regime of small sphere separations.

Figure 8(a) shows an overview of the relative difference $\delta$ between the extinction efficiency of the double-sphere and the single sphere, defined in Eq. (8) as a function of *l* on a linear scale. It can be observed that the difference between the extinction efficiency of the single sphere and the double-sphere significantly decreases as *l* increases. A different view of the data is presented in Fig. 8(b), which shows the data in Fig. 8(a) on a double-log (log–log) scale. We see that the relative difference $\delta$ decays like a power-law for large distances. The dashed lines in Fig. 8(b) are are not least-square fits, but only intended to guide the eye. They represent power-law decays of $\delta =0.013/l^{0.4}$ for E-polarized data and $\delta =0.017/l^{0.4}$ for H-polarized data. That we might expect a power-law decay at large distances can be motivated on the basis of the Born-series expansion of the scattered electric field^19^ and is discussed in more detail below in the Discussion section. A more detailed view of the data in Fig. 8(a) and (b) for small sphere separations is presented in Fig. 8(c) on a semi-log scale. In this view of the data we see that, for small distances *l* between the spheres, $\delta$ decays exponentially with a cross-over to power-law decay (see Fig. 8(b)) at $l\approx 0.7\,\mu$m for H-polarization and $l\approx 1\,\mu$m for E-polarization. The exponential decay at small sphere separation is understood on the basis of the fact that the whispering-gallery modes of the spheres generate a near field that has the character of an exponentially decaying evanescent field beyond the surface of the spheres. This leakage can clearly be identified visually in Fig. 6. Since evanescent fields typically decay on the scale of a wavelength, this explains the short range of the exponential decay observed in Fig. 8(c).

### Qext of sphere arrays

**Figure 9:**
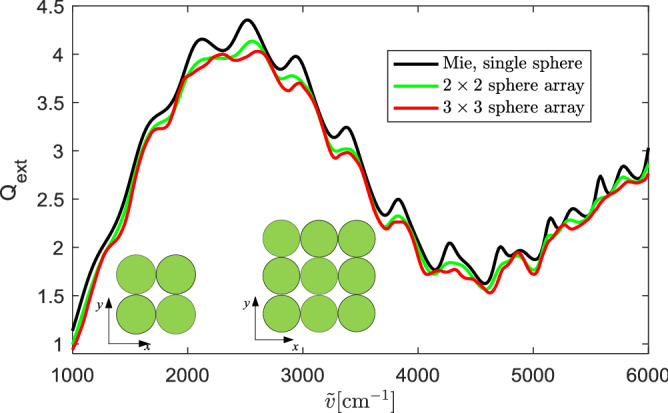
Extinction efficiency of the $2\times 2$ and $3\times 3$ sphere arrays compared with the exact Mie solution of the single sphere. The spheres have a diameter of $5.5\, \mathrm {\upmu }$m and a refractive index of 1.49.

We next simulate targets that contain more spheres. The extinction efficiency of a $2\times 2$ sphere array and that of a $3\times 3$ sphere array are calculated. The spheres are distributed in the *x*–*y* plane, as shown in Fig. 9. In both sphere arrays, the spheres are in touch with their adjacent spheres. Each sphere has a diameter of $5.5\, \mathrm {\upmu }$m and a refractive index of 1.49. The targets are illuminated by a plane wave propagating along the $-z$ direction with an *x*-polarized incident electric field. The simulation results of the sphere arrays are compared with the Mie solution of the single sphere in Fig. 9. Compared with the double-sphere, the difference between $Q_\mathrm{ext}$ of sphere arrays and the Mie result of the single sphere is larger, and increasing the number of spheres increases this difference. The relative difference $\delta$ (defined in Eq. (8)) between $Q_\mathrm{{ext}}$ of a $2\times 2$ sphere array and the single sphere is 0.04. For the $3\times 3$ sphere array, $\delta$ increases to 0.06. Nevertheless, the wiggles in $Q_\mathrm{ext}$ of sphere arrays still agree well with those of the single sphere.

**Figure 10:**
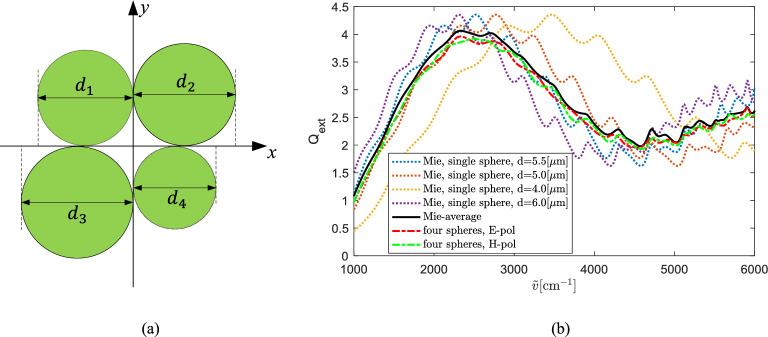
(**a**) Sphere array containing spheres of different sizes. (**b**) Extinction efficiency of the sphere array in Fig. 10(a) compared with the exact Mie solution. The spheres have a refractive index of 1.49.

We also calculated the scattering of targets that contain spheres of different sizes, as shown in Fig. 10(a). The diameters of the four spheres in the *x*–*y* plane are $d_1=5.0\, \mathrm {\upmu }$m, $d_2=5.5\, \mathrm {\upmu }$m, $d_3=6.0\, \mathrm {\upmu }$m, and $d_4=4.0\, \mathrm {\upmu }$m, respectively. The refractive indexes of the spheres are all 1.49. The incident plane wave is propagating along the $-z$ direction. The extinction efficiencies of the four-spheres array under the two different polarizations are shown in Fig. 10(b). For comparison, the Mie results of the single spheres with different diameters together with the average

9 $$\begin{aligned} \mathrm{Mie\text {-}average} \left( \tilde{v}\right) =\frac{\sum _{i=1}^{4}{Q^\mathrm{ext}_{i}g_i}\left( \tilde{v}\right) }{\sum _{i=1}^{4}g_i} \end{aligned}$$

of the Mie results are also plotted in Fig. 10(b). In Eq. (9), $Q^\mathrm{ext}_{i}$ is the extinction efficiency of the sphere with a diameter of $d_i$, and $g_i=\pi d_i^2/4$ is the geometric cross section of the *i*th sphere. Our simulation results show that the wiggles of the extinction efficiency of the four spheres agree with that of the average of the Mie results. Compared with the single sphere, $Q_\mathrm{ext}$ of the four spheres is smoother, i.e, the ripples are less noticeable.

### Experimental results

In order to verify our numerical studies and simulations, we measured the FTIR spectrum of PMMA microspheres and compared the experimental results with the numerical results. The PMMA microspheres have an average diameter of $5.5\, \mathrm {\upmu }$m.

The PMMA samples do not have an ideal real constant refractive index, but a complex refractive index that depends on the wavenumber^20,21^. Moreover, in our FTIR experiments, the incident field is not a plane wave propagating in the forward direction, but an infrared beam which can be considered as the superposition of plane waves propagating in a range of directions^22^. For a better comparison with experimental results, we calculate the scattering of a double-sphere that has a complex refractive index as published in^20,21^. The double-sphere is illuminated by normal and oblique incident plane waves. The double-sphere is arranged along the *x*-direction (see Fig. 2). The diameter of both spheres is $5.5\, \mathrm {\upmu }$m. The distance between the spheres is *l*. As indicated by the blue arrow in Fig. 2, the electric field of the incident plane wave is *y*-polarized and propagates in the *x*–*z* plane. The angle between the incident direction and the $-z$ direction is $\theta$.

**Figure 11:**
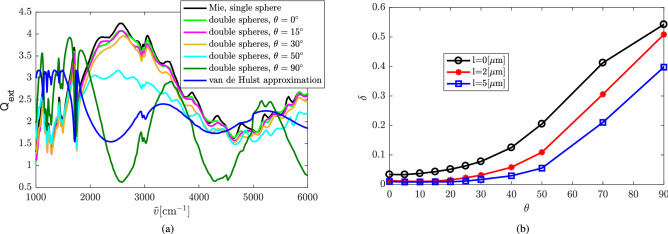
(**a**) Extinction efficiency of two touching PMMA spheres for different incident angles. The spheres have a diameter of $5.5\, \mathrm {\upmu }$m and a frequency-dependent refractive index as published in^20,21^. (**b**) Relative difference between the extinction efficiency of the double-sphere and the single sphere as a function of $\theta$.

Figure 11(a) shows the extinction efficiency of two touching PMMA spheres ($l=0$) under different incident directions and the exact Mie solution of the single PMMA sphere. From the results we observe that the coupling of the two PMMA spheres hardly affects the wiggles of the extinction efficiency when $\theta <30^\circ$. For large incident angles ($\theta =50^\circ , 90^\circ$), the difference between $Q_\mathrm{ext}$ of double PMMA spheres and the Mie result of the single PMMA sphere is very large. Especially when the incident direction is along the longitudinal direction of the double-sphere ($\theta =90^\circ$), the wiggles in $Q_\mathrm{ext}$ of the double-sphere and that of the single sphere are completely different. In fact, Fig. 11(a) shows that the frequency of the wiggle in the double-sphere case at $\theta =90^\circ$ is twice the wiggle frequency for a single sphere. This is immediately understood considering that the optical path length of rays traversing the double-sphere at $\theta =90^\circ$ is twice the optical path length of rays traversing a single sphere. Thus, the phase shift experienced by a light ray traversing the double-sphere is twice the phase-shift experienced by a ray traversing a single sphere. Therefore, since $\rho$ in (7) is the phase-shift of the central ray through the sphere, we replace $\rho \rightarrow 2\rho$ in (7) and obtain the analytical van de Hulst result for $Q_\mathrm{ext}$ at $\theta =90^\circ$ according to

10 $$\begin{aligned} Q_\mathrm{ext} \approx 2 - \frac{2}{\rho } \sin (2\rho ) + \frac{1}{\rho ^2} [1-\cos (2\rho )]. \end{aligned}$$

The result (10) is shown as the blue line in Fig. 11(a). Qualitatively, it agrees well with the simulation result for the double-sphere at $\theta =90^\circ$. Compared to the van de Hulst result for a single sphere (7), we see that (10) has exactly twice the frequency as a function of wavenumber $\tilde{\nu }$ than (7). This explains quantitatively the doubling of the wiggle frequency for the double-sphere with $\theta =90^\circ$.

Figure 11(b) shows the relative difference $\delta$ between $Q_\mathrm{ext}$ of the single sphere and that of double PMMA spheres with different distances as a function of $\theta$. Again, the effect of the coupling of the spheres gets stronger as $\theta$ increases from $0^\circ$ to $90^\circ$. The coupling effect gets weaker as the distance of the double-spheres gets larger. According to typical experimental setups in FTIR spectroscopy, the maximum incident angle of most of the rays of the incident beam is less than $25^\circ$. Therefore, the coupling of the PMMA spheres does not dramatically affect the Mie wiggles of the spectra. This is an important observation, since some correction algorithms in widespread use^8,9^ are based on the Mie wiggles.

**Figure 12:**
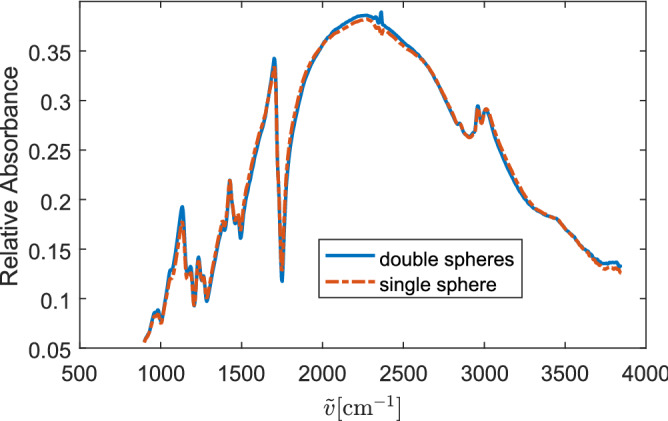
Measured absorbance spectra of PMMA samples.

We now turn to the measured absorbance spectra of the PMMA samples. The spectra of two touching PMMA spheres and that of an isolated PMMA sphere are shown in Fig. 12. Since our experiments were performed with a thermal source, the incident radiation is unpolarized. Therefore, the measured spectra are the average of E- and H-polarization. Figure 12 shows that the absorbance spectra of these two samples have the same wiggles. Thus, the experimental results are in line with our above predictions.

## Discussion

From our numerical experiments, several conclusions may be drawn. First, the extinction efficiency of two touching PMMA spheres is only slightly different from that of a single sphere when the incident direction is along the transversal plane (see Fig. 2). The wiggles of the extinction efficiency of the two touching spheres are the same as the Mie wiggles of the single sphere. The coupling of the double-sphere only significantly affects the ripples (resonances) in $Q_\mathrm{ext}$ when the polarization of the incident field matches the type of resonance. The significant differences between $Q_\mathrm{ext}$ of the double-sphere and the single-sphere appear near electric-type resonances when the incident electric field is along the longitudinal direction of the double-sphere, and near magnetic-type resonances when the incident magnetic field is along the longitudinal direction of the double-sphere. This phenomenon can be observed more clearly for spheres that have a larger refractive index, which results in sharper resonances. When considering wave functions at wavenumbers corresponding to the sharp ripples, we can clearly see the corresponding standing waves inside of the single sphere. This whispering-gallery mode structure, which is characteristic of single spheres, may be suppressed when another sphere touches the surface guiding the standing wave^7^. As we observed, both whispering-gallery mode structures of the two touching spheres are suppressed when they are located in the same plane in which the whispering-gallery wave is located.

We also observe that when the distance between the double-spheres gets larger, the already small coupling effect of the spheres decreases dramatically. For example, when the double-spheres with a diameter of $5.5\, \mathrm {\upmu }$m and $n=1.49$ are approximately one radius apart, the relative difference $\delta$ between the extinction efficiency of the double-sphere and that of the single sphere is less than 0.02 for both E- and H-polarization.

For $2\times 2$ and $3\times 3$ sphere arrays, the extinction efficiency still displays the same wiggles as the Mie solution of the single sphere. If the spheres have different sizes, the wiggles of the extinction efficiency agree with the average of the Mie solutions of the single spheres, i.e., the total extinction cross section divided by the total geometric cross section. Compared with the double-spheres, the ripples of sphere arrays are more suppressed. For sphere arrays, the coupling of the spheres affects all types of resonances since the spheres are arranged in both orthogonal directions on the plane perpendicular to the incident wave. Moreover, if the spheres have different sizes, $Q_\mathrm{ext}$ of the average of the Mie solution is smoother than $Q_\mathrm{ext}$ of each single sphere. However, the wiggle structure is still conserved.

The extinction efficiency of the double PMMA sphere changes as a function of the angle between the incident direction and the normal direction of the plane where the sample is located. The larger this angle, the stronger the effect of the coupling of the two spheres on the extinction efficiency. When the incident direction is along the longitudinal direction of the double-sphere, $Q_\mathrm{ext}$ has twice the wiggle frequency compared to a single sphere and is therefore completely different. This was explained quantitatively in the Results section. However, this situation is not encountered in an infrared microscope, unless spherical samples overlap with each other. Since the maximum incident angle of the incident radiation in FTIR spectroscopy is typically less than $25^\circ$, the effect of coupling on the ripple structure is small and can hardly be measured.

For biological samples the ripples are normally suppressed and not visible^11^, while, for monodisperse samples^12^, the wiggle structure is pronounced and consistent with the Mie model^11^. Therefore, the results of this study support the validity of the ME-EMSC method for the correction of absorbance spectra of biological cells that are in a cluster or tissue. The ME-EMSC algorithm uses the van de Hulst approximation for $Q_\mathrm{{ext}}$ of a sphere, which only considers the Mie-wiggle structure and does not consider the Mie ripples. Our results show that the ME-EMSC method is valid for samples that contain more than one spherical scatterer since the coupling of these scatterers hardly affects the Mie wiggles.

Recently, the ripple structure in the spectrum was shown to have the potential to be used to determine both the radius and the frequency-dependent refractive index of PMMA spheres^7^. In the future, the spectrum of a double-sphere may also be used in this application. For example, based on the characteristic of the effect of the coupling of double-spheres on the ripples, we may extract different types of ripples under different polarizations. This would help to improve the accuracy of the determination of the locations and types of the ripples.

The smallness of the coupling effect between two touching spheres and the rapid decay of the coupling effect with distance can be understood both qualitatively and quantitatively on the basis of the Born series solution of the scattering problem^19^. Given a scatterer with dielectric constant $\epsilon (\vec r)$, the electric field of the scattering system satisfies the Lippmann–Schwinger equation^19^

11 $$\begin{aligned} \mathbf {E}(\mathbf {r}) = \mathbf {E}^i (\mathbf {r})+ k^2 \int G(\mathbf {r},\mathbf {r'}) [\epsilon (\mathbf {r'})-1] \mathbf {E}(\mathbf {r'}) d^3 \mathbf {r'}, \end{aligned}$$

where $\mathbf {E}^i (\mathbf {r})$ is the incident electric field, $G(\mathbf {r},\mathbf {r'})$ is the analytically known, transverse electromagnetic Green function^19^, $\epsilon (\mathbf {r})$ is the dielectric constant of the scatterer, and the integration is over the volume of the scatterer. Equation (11) can formally be solved iteratively according to

12 $$\begin{aligned} \mathbf {E}_m (\mathbf {r})&= \mathbf {E}^i (\mathbf {r})+ k^2 \int G(\mathbf {r},\mathbf {r'}) [\epsilon (\mathbf {r'})-1] \mathbf {E}_{m-1}(\mathbf {r'}) d^3 \mathbf {r'}, \nonumber \\ m&=1,2,\ldots , \end{aligned}$$

where $\mathbf {E}_0 (\mathbf {r}) =\mathbf {E}^i (\mathbf {r})$. Let us denote by $\epsilon _1(\mathbf {r})$ the dielectric constant of the first sphere and by $\epsilon _2(\mathbf {r})$ the dielectric constant of the second sphere. Then,

13 $$\begin{aligned} \epsilon _1(\mathbf {r}) \epsilon _2(\mathbf {r}) =0, \end{aligned}$$

since, although touching, the two spheres have no overlap. Let us also split the incident field according to

14 $$\begin{aligned} \mathbf {E}^i (\mathbf {r})= \mathbf {E}^{i,1} (\mathbf {r})+ \mathbf {E}^{i,2} (\mathbf {r}), \end{aligned}$$

where $\mathbf {E}^{i,1} (\mathbf {r})$ is the incident field illuminating sphere 1 and $\mathbf {E}^{i,2} (\mathbf {r})$ is the incident field illuminating sphere 2. Then, for $m=1$ (first Born approximation) the total field generated by the two touching spheres is

15 $$\begin{aligned}{}&\mathbf {E}_1 (\mathbf {r})= \mathbf {E}^{i,1} (\mathbf {r})+ \mathbf {E}^{i,2} (\mathbf {r})+ \nonumber k^2 \int G(\mathbf {r},\mathbf {r'}) [\epsilon _1(\mathbf {r'})+ \epsilon _2(\mathbf {r'})-1] \mathbf {E}_0(\mathbf {r'}) d^3 \mathbf {r'}, \nonumber \\&= \mathbf {E}^1 (\mathbf {r})+ \mathbf {E}^2 (\mathbf {r}), \end{aligned}$$

where $\mathbf {E}^1 (\mathbf {r})$ and $\mathbf {E}^2 (\mathbf {r})$ are the electric fields generated by spheres 1 and 2 individually, since, because of (13), the integral in (15) splits into two disjointed parts. This also implies that the total scattering amplitude of the touching two-spheres system is the sum of the scattering amplitudes of the two individual spheres, and, in particular, that the total scattering amplitude in forward direction is simply the sum of the scattering amplitudes of the two individual spheres. According to the optical theorem^19^, which states that the total cross section is proportional to the imaginary part of the total amplitude in forward direction, the total cross section of the two-spheres system is now exactly twice the scattering cross section of a single sphere, and if normalized by twice the geometric cross section of a single sphere, we obtain, in first-order Born approximation, the exact relation $Q_\mathrm{ext} (\text {double-sphere})= Q_\mathrm{ext} (\text {single sphere})$. This supports our finding that the coupling between the two spheres of a two-spheres system is small.

Since there is no coupling between the two spheres in first-order Born approximation, all observed coupling effects must be due to the second and higher orders of the Born series (12). The second order of the Born series is obtained by iterating (12) once, which produces coupling terms of the form

16 $$\begin{aligned} \int d^3 \mathbf {r'} \int d^3 \mathbf {r''} G(\mathbf {r},\mathbf {r'}) G(\mathbf {r'},\mathbf {r''}) \epsilon _1(\mathbf {r'}) \epsilon _2(\mathbf {r''}) \mathbf {E}_0(\mathbf {r'}) \mathbf {E}_0(\mathbf {r''}) , \end{aligned}$$

which now, clearly, couple the spheres, involving the Green function $G(\mathbf {r'},\mathbf {r''})$, which propagates radiation from one sphere to the other. Since, to leading order,

17 $$\begin{aligned} G(\mathbf {r'},\mathbf {r''}) \sim \exp (ik|\mathbf {r'}-\mathbf {r''}|)/ |\mathbf {r'}-\mathbf {r''}| , \end{aligned}$$

we see that, especially with increasing distance, the coupling vanishes according to a power-law, and is additionally suppressed by the oscillating term $\exp (ik|\mathbf {r'}-\mathbf {r''}|)$. That we see a power-law different from $-1$ in our simulations may, in part, be due to the fact that we average over a large range of wavenumbers when computing the difference $\delta$.

The Born series supports our intuition that the coupling effect of two spheres is due to rescattering of radiation by the other sphere into the forward direction. The probability of this two-step process to occur is small since it involves the product of the small amplitude that scattering by one of the spheres out of the forward direction occurs in the first place and the small amplitude that this scattered radiation is then redirected into the forward direction by the other sphere. Since it involves the product of two small amplitudes, the total probability of two-step rescattering, and therefore the coupling effect, is small.

As already discussed in “Results” section, the origin of the exponential decay of the coupling at very small distances is due to the coupling of the two spheres via evanescent waves that leak beyond the surfaces of the two spheres. This is the exponential regime which cannot be explained via a low-order Born series. Since the evanescent waves are strongest at the Mie resonances (i.e., the whispering-gallery modes) a proper resonance theory^19^ is needed to explain this regime quantitatively. This, however, is beyond the scope of this paper.

## Conclusion

In this work, we employed the SIE method, a scheme based on the full numerical solution of Maxwell’s equations, to study the effects of the coupling of dielectric spherical particles on signatures in infrared microspectroscopy. We showed that the wiggle structure of the extinction spectra is hardly affected by the coupling of the spheres unless the angle between the incident direction of the source and the plane where the spheres are located is large. This conclusion is also verified by the experimental results, where the incident radiation contains both normal and oblique incidences due to the use of a high numerical aperture in the infrared microscope. The results show that the current correction methods for infrared absorbance spectra, such as ME-EMSC, are valid for samples that contain multi-cell scatterers in the plane of the substrate. Our findings are further supported by the practical experience with ME-EMSC, which has been successfully applied to cells in close vicinity to other cells^23^. In addition, we showed that the ripple structure is significantly affected by the coupling of double-spheres when the polarization of the incident wave matches the resonance type of that ripple. Moreover, the ripple structure is more affected by the coupling of spheres in sphere arrays.

While the above conclusions refer to cells and carpets of cells in the plane of the substrate, the situation is completely different for overlapping cells, cells arranged on top of each other, or for multi-layered carpets of cells. In this case, both our analytical van-de-Hulst calculations as well as our simulations with oblique incidence indicate that strong coupling effects should occur resulting in strong scattering effects that, e.g., have the ability to change the frequency of Mie wiggles on which some spectral correction methods, such as ME-EMSC^9^, are based. Due to the difficulty of performing controlled experiments on stacked cells, or even stacked or overlapping micro-spheres, we are currently unable to present experimental evidence supporting our theoretical predictions concerning out-of-plane cells. Thus, we leave the experimental test of our out-of-plane predictions for future experimental work. Nevertheless, the results of this study, focused on in-plane cell arrangements, provide us with a deeper understanding of the properties of coupling effects of targets consisting of strings and arrays of spheres, and provide guidance in the proper extraction of information from samples of more complex structure and composition.

## Data Availability

The datasets generated during and/or analysed during the current study are available from the corresponding author on reasonable request.
